# Supplementary material for: A study on limited pre-sale strategy with consideration of consumer regret
Source: PLoS One. 2023 May 3;18(5):e0285052. doi: 10.1371/journal.pone.0285052 (PMC10155970; doi:10.1371/journal.pone.0285052)
Supplement: S1 Appendix — (DOCX) [file pone.0285052.s001.docx]

Appendix A

**Appendix A.1 Proof of Theorem 1.**

Compare the profit function for strategy 1 and Strategy 2:

1. When $C\leq\frac{1}{2}, {\pi_{1}}^{*}-{\pi_{2}}^{*}=V_{L}C-\frac{1}{2}V_{H}<0$, Strategy 2 is better;
2. When $\frac{1}{2}<C\leq\frac{V_{H}}{2V_{L}}, {\pi_{1}}^{*}-{\pi_{2}}^{*}=\left( V_{L}-V_{H} \right)C<0$, Strategy 2 is better;
3. When $C>\frac{V_{H}}{2V_{L}}, {\pi_{1}}^{*}-{\pi_{2}}^{*}=V_{L}C-\frac{1}{2}V_{H}>0$, Strategy 1 is better;

**Appendix A.2 Proof of Theorem 2.**

Through the analysis of consumer behavior when the retailer adopts strategy 3:

1. When $C\leq a/2$, the capacity of the retailer can only satisfy all the needs of the high-valued rational consumers, and the retailer can not pre-sell, and the maximum profit is $V_{H}C$;
2. When $a/2<C\leq1/2$, the capacity of the retailer can meet all the demand at the present sale stage and part of the demand at the pre-sale stage. In order to make the profit bigger, the high-valued consumers who arrive at the pre-sale stage buy at the present sale stage, when the optimal pre-sale quantity is $C-\frac{a}{2}$, the profit function is $V_{L}\left( C-\frac{a}{2} \right)+V_{H}\frac{a}{2}$;
3. When $C>1/2$, retailers can meet all the needs of consumers. At this time if not limited pre-sale, in the pre-sale stage to reach the high-value consumers will buy in advance, damage part of the profit. Therefore, at this time also limited pre-sale, forced to wait until the current sale stage to buy. The optimal pre-sale quantity is $\frac{2-a}{4}$, and the profit function is $V_{L}\frac{2-a}{4}+V_{H}\frac{a}{2}$.

**Appendix A.3 Proof of Theorem 3.**

Through the analysis of consumer behavior when the retailer adopts strategy 4:

1. When $C\leq1/2$, the capacity of the retailer can only meet the needs of all high-valued consumers, at this time not pre-sale, the maximum profit is $V_{H}C$;
2. When $1/2<C\leq3/4$, the capacity of the retailer can meet all the demand at the present sale stage and part of the demand at the pre-sale stage, at this time the limited pre-sale. In order to make the profit bigger, the high-valued consumers who arrive at the pre-sale stage buy at the present sale stage, when the optimal pre-sale quantity is $C-\frac{1}{2}, \theta=4C-2$, so the profit function is $V_{L}\left( C-\frac{1}{2} \right)+\frac{V_{H}+\beta\left( 4C-2 \right)V_{L}}{1+\beta\left( 4C-2 \right)}\frac{1}{2}$;
3. When $C>3/4$, the capacity of the retailer can meet all the needs of consumers, for the same reason, in order to ensure greater profits, limited pre-sale, the optimal pre-sale volume is 1/4, $\theta=1$, the profit function is $V_{L}\frac{1}{4}+\frac{V_{H}+\beta V_{L}}{1+\beta}\frac{1}{2}$.

**Appendix A.4 Proof of Theorem 4.**

Combining theorem 2 and theorem 3:

1. When $C\leq\frac{a}{2}, {\pi_{3}}^{*}={\pi_{4}}^{*}=V_{H}C$, not pre-sold at this time;
2. When $\frac{a}{2}<C\leq\frac{1}{2}, {\pi_{3}}^{*}-{\pi_{4}}^{*}=V_{L}\left( C-\frac{a}{2} \right)+V_{H}\frac{a}{2}-V_{H}C<0$, the product is not pre-sold;
3. When $\frac{1}{2}<C\leq\frac{3}{4}, {\pi_{3}}^{*}-{\pi_{4}}^{*}=V_{L}\frac{2-a}{4}+V_{H}\frac{a}{2}-\left( V_{L}\left( C-\frac{1}{2} \right)+\frac{V_{H}+\beta\left( 4C-2 \right)V_{L}}{1+\beta\left( 4C-2 \right)}\frac{1}{2} \right)$. Let ${\pi_{3}}^{*}-{\pi_{4}}^{*}>0, a>a_{1}=\frac{2(V_{H}-2V_{L}+2CV_{L}+2\beta V_{L}-8C\beta V_{L}+8C^{2}\beta V_{L})}{(1-2\beta+4C\beta)(2V_{H}-V_{L})}$. And because $0<a_{1}<1$, strategy 3 is better when $a>a_{1}$. Otherwise, Strategy 4 is better;
4. When $C>\frac{3}{4}, {\pi_{3}}^{*}-{\pi_{4}}^{*}=V_{L}\frac{2-a}{4}+V_{H}\frac{a}{2}-\left( V_{L}\frac{1}{4}+\frac{V_{H}+\beta V_{L}}{1+\beta}\frac{1}{2} \right)$. Let ${\pi_{3}}^{*}-{\pi_{4}}^{*}>0, a>a_{2}=\frac{2V_{H}-V_{L}+\beta V_{L}}{(1+\beta)(2V_{H}-V_{L})}$. And because of $0<a_{2}<1$, strategy 3 is better when a > A, otherwise strategy 4 is better.

**Appendix A.5 Proof of Theorem 5.**

Through the analysis of consumer behavior when $\gamma\leq\frac{-1-\beta\mu}{\beta(-1+\mu)}$ and the retailer adopts strategy 5 or strategy 6:

1. When $C\leq\frac{a}{4}$, Strategy 5 and strategy 6 are not pre-sold;
2. When $a/4<C\leq1/4$, strategy 5 is pre-sold at an unlimited premium, and strategy 6 is not pre-sold. Because ${\pi_{5}}^{*}-{\pi_{6}}^{*}=\frac{1}{4}(a-4C)(V_{H}-V_{L})<0$, so select strategy 6, do not pre-sell;
3. When $C>1/4$, both strategy 5 and strategy 6 are pre-sold unlimited. ${\pi_{5}}^{*}-{\pi_{6}}^{*}=\frac{(-3+a(3+(-1+4C)\beta))(V_{H}-V_{L})}{4(3+(-1+4C)\beta)}$.Let ${\pi_{5}}^{*}-{\pi_{6}}^{*}>0 , a>a_{3}=\frac{3}{3-\beta+4C\beta}$. And because $0<a_{3}<1$, so when $a>a_{3}$, the retailer uses strategy 5; otherwise strategy 6 is better.

**Appendix A.6 Proof of Theorem 6.**

Through the analysis of consumer behavior when $\gamma>\frac{-1-\beta\mu}{\beta(-1+\mu)}$ and the retailer adopts strategy 5 or strategy 6:

1. When $C\leq\frac{a}{4}$, Strategy 5 and strategy 6 are not pre-sold;
2. When $a/4<C\leq1/4$, strategy 5 does not pre-sell at an unlimited premium, and strategy 6 does not pre-sell. Because of ${\pi_{5}}^{*}-{\pi_{6}}^{*'}=\frac{1}{4}(a-4C)(V_{H}-V_{L})<0$, so retailers choose strategy 6, not pre-sale;
3. When $1/4<C\leq\left( 3+a \right)/4$, both strategy 5 and strategy 6 were pre-sold unlimited. Because ${\pi_{5}}^{*}-{\pi_{6}}^{*'}=\frac{(-2+a+a^{2}+a(-1+4C)\beta)(V_{H}-V_{L})}{4(2+a+(-1+4C)\beta)}$, if we make ${\pi_{5}}^{*}-{\pi_{6}}^{*}>0$, we get $\beta>\beta_{1}=\frac{2-a-a^{2}}{a(-1+4C)}$. Because $\beta_{1}>0$, so when $\beta>\beta_{1}$, the retailer adopts strategy 5, otherwise, it adopts strategy 6.
4. When $C>\left( 3+a \right)/4$, both strategy 5 and strategy 6 are pre-sold unlimited. Because ${\pi_{5}}^{*}-{\pi_{6}}^{*'}=\frac{(-1+a+a\beta)V_{H}-(2+3\beta+2a(1+\beta)-4C(1+\beta))V_{L}}{4(1+\beta)}$, if we make ${\pi_{5}}^{*}-{\pi_{6}}^{*}>0$, we get $\beta>\beta_{2}=\frac{-(-1+a)V_{H}+2(1+a-2C)V_{L}}{aV_{H}+(-3-2a+4C)V_{L}}$. Because $\beta_{2}>0$, so when $\beta>\beta_{1}$, the retailer adopts strategy 5, otherwise, it adopts strategy 6.

**Appendix A.7 Proof of Theorem 7.**

Combining theorem 1,4,5 and 6:

1. First of all, the comparison between no pre-sale and discount pre-sale shows that: when $C\leq1/2$, no pre-sale is better. So just compare the no-pre-sale to the premium pre-sale: first, the no-pre-sale to the premium pre-sale:
2. If $\gamma\leq\frac{-1-\beta\mu}{\beta(-1+\mu)}$: when $C\leq1/4$, do not pre-sell;

when $1/4<C\leq1/2$ and $a>a_{3}$, it is not pre-sold as compared with strategy 5. Because $\pi^{*}-{\pi_{5}}^{*}=-\frac{1}{4}(a-4C)(V_{H}-V_{L})>0$, so it is not pre-sold at this time;

when $1/4<C\leq1/2$ and $a\leq a_{3}$. Because $\pi^{*}-{\pi_{6}}^{*}=\frac{(-1+4C)(3+4C\beta)(V_{H}-V_{L})}{4(3+(-1+4C)\beta)}>0$, it is not pre-sold;

1. If $\gamma>\frac{-1-\beta\mu}{\beta(-1+\mu)}$: when $C\leq1/4$, do not pre-sell;

when $1/4<C\leq1/2$ and $\beta>\beta_{1}$, it is not pre-sold as compared with strategy 5. Because $\pi^{*}-{\pi_{5}}^{*}=-\frac{1}{4}(a-4C)(V_{H}-V_{L})>0$, so it is not pre-sold at this time;

when $1/4<C\leq1/2$ and $\beta\leq\beta_{1}$. Because $\pi^{*}-{\pi_{6}}^{*'}=\frac{(-1+4C)(2+a+4c\beta)(V_{H}-V_{L})}{4(2+a+(-1+4c)\beta)}>0$, it is not pre-sold;
