# Supplementary material for: A study on limited pre-sale strategy with consideration of consumer regret
Source: PLoS One. 2023 May 3;18(5):e0285052. doi: 10.1371/journal.pone.0285052 (PMC10155970; doi:10.1371/journal.pone.0285052)
Supplement: Attachment — Submitted filename: Response to Reviewers.docx [file pone.0285052.s003.docx]

Dear Editor and Reviewers,

Thank you so much for taking the time to review this manuscript. We appreciate your comments and suggestions. According to the instructions in your letter, we have uploaded the revisions, all of which are highlighted using the change mode in Word. We enclose our responses to the comments made by the academic editor and two commentators. Comments from academic editors are reproduced in italics, and our responses are given directly in a different color (red) . Thanks Again!

**Academic Editor**

*Q1: First, you need to properly adjust the literature source.*

**Answer1:** Thanks for your suggestion, we agreed and updated the content. We rearranged the existing research reviews and added new citations. Amend it as follows:

(1) Line 91-135, page 3-4:

Early research on pre-sale models focused on the service industry, commonly pre-sales of various types of tickets. Jinhong X and Steven M showed that offering pre-sales is beneficial for firms, especially when consumers are uncertain about the value of the product [7]. Yu M et al. demonstrated that pre-sale may be advantageous when there is a large number of consumers and a high degree of individual valuation diversification [8]. Li C et al explored the pre-sale decision in a pre-sale model when both short-sighted and strategic consumers are present [9]. Then Li H and Qi ES comparatively studied pre-selling and non-pre-selling strategies so as to give the conditions for retailers to implement pre-selling strategies [10]. However, there is little literature on limited pre-sale. After that, Chen J and Bell PC studied the demand uncertainty of fixed capacity and limited product choice for fugitive products [11]. Zhang M et al explored the limited pre-sale model for new products on flash sale platforms [12].Ji GJ and so on introduced the reference price effect in the pre-sale model, thus obtained the retailer's optimal pre-sale decision [13], and then continued to study the ref-erence price effect on the limited pre-sale strategy [14].

(2) Line 137-153, page 4:

Expected regret reflects a combination of regret, depression, disappointment, remorse, and self-blame that will be experienced by not buying the limited product [15,16,17], coupled with the higher perceived value of the product that comes with a production-based limited [18] and the stronger motivation for consumers to own the product [19]. Thus, compared to sales-based limits, production-based limits are more likely to induce strong feelings of expected elation and expected regret. In the area of consumer regret research, Zen Z and Zheng Y considered the high price that consumers would face if they bought immediately after the full-price period and the stock-out that they would face if they waited until the liquidation period [20]. Then, Adida E and ÖZER Ö, on the basis of Zen Z and Zheng Y, considered the effect of anticipatory regret behavior on firms' pricing strategy choice in the competitive market environment, and emphasized the advantage of price reduction strategy [21]. Chen FJ and Guan ZZ studied the impact of expected regret on return strategies when consumers' valuations are uncertain [22]. Zhou JH et al analyzed the impact of high price regret and out-of-stock regret on the pricing strategy of enterprises [23]. Shi BL and other considerations when consumers pre-purchase regret behavior does not provide returns, returns no longer on sale and return re-sale of pre-sale strategy [24].

***Q2:*** *Second, you must correct the format of the reference and possibly other relevant parts.*

**Answer2:** Thank you for your suggestions, we agree and have updated the content. We have revised “introduction”. Specific modifications are as follows:

(1) Line 632-906, page 18-19: The first paragraph has been revised and 4 citations have been added.
1. Song JS, Paul H, Zipkin. Newsvendor problems with sequentially revealed demand information. Naval Research Logistics. 2012:59(8): 601-612.
2. Christopher S, Tang, Kumar R, Aydin A, Jihong O. The Benefits of Advance Booking Discount Programs: Model and Analysis. Management Science. 2004;50(4) : 465-478.
3. Mao ZF, Dong ZC, Liu W. Comparative research on joint strategy of advance selling and buy-back to conventional products and new products. Computer integrated manufacturing systems. 2017;23(04) : 867-873.
4. Wendy W. Moe,Peter S. Fader. Fast-track: Article using advance purchase orders to forecast new product sales.Marketing science.2002;21(3) : 347-364.
5. Volker N,Martin P,Frank R.Advance-purchase discounts as a price discrimination device. Journal of Economic Theory.2011;146(1) : 141-162.
6. Shugan SM,Xie J. Advance selling for service.California Management Review.2004;46(3) : 37-5.
7. Jinhong X and Steven M. Shugan. Electronic Tickets, Smart Cards, and Online Prepayments: When and How to Advance Sell. Marketing Science. 2001;20(3) : 219-243.
8. Yu M, Kapuscinski R, Ahn HS. Advance Selling: Effects of Interdependent Consumer Valuations and Seller’s Capacity. Management Science. 2015; 61(9):2100-2117.
9. Li C, Zhang F. Advance demand information, price discrimination,and preorder strategies. Manufacturing & Service Operations Management.2013;15(1) : 57-71.
10. Li H, Qi ES. Advance Sellingin the Presence of Uncertainty Market Size. Chinese Journal of Management Science.2017;25(02):50-56.
11. Chen J, Bell PC. Enhancing revenue by offering a flexible product option. International Transactions in Operational Research. 2017;24(4): 801–820.
12. Zhang M,Cheng T,Du J. Advance selling of new products to strategic consumers on flash sale platforms. International Journal of Logistics: Research and Applications.2018;21(3) : 318-331.
13. Ji GJ, Sun ZF. Advance selling with reference price effect and consumer heterogeneity. Systems Engineering-Theory & Practice. 2018; 38(12): 3059-3070.
14. Ji GJ, Sun ZF ,Yang GY, Zeng QY, et al. Advance selling with consumer reference price effect and limited advance sales. Journal of Industrial Engineering and Engineering Management. 2021;35(04): 178-189.
15. Bagozzi,Utpal M. Dholakia,Suman B. How effortful decisions get enacted: The motivating role of decision processes, desires, and anticipated emotions. Journal of Behavioral Decision Making. 2003;16(4):273－295.
16. Perugini M,Bagozzi RP. The role of desires and anticipated emotions in goal-directed behaviours: Broadening and deepening the theory of planned behavior. The British Journal of Social Psychology.2001;40(1):79－98.
17. Proksch M, Orth UR, Cornwell TB. Competence enhancement and anticipated emotion as motivational drivers of brand attachment. Psychology & Marketing. 2015;32(9) : 934－949.
18. Jang WE, Ko YJ, Morris JD,et al. Scarcity message effects on consumption behavior: Limited edition product considerations. Psychology & Marketing.2015;32(10):989－1001.
19. Tian KT, Bearden WO, Hunter GL. Consumers’need for uniqueness: Scale development and validation. Journal of Consumer Research. 2001; 28(1):50－66.
20. Zen Z, Zheng Y. Markdown or Everyday Low Price? The Role of Behavioral Motives. Management Science. 2016; 62(2): 326-346.
21. Adida E, ÖZER Ö. Why Markdown as a Pricing Modality?. Management Science. 2019; 65(5):2161-2178.
22. Chen SJ, Guan ZZ. Return policy for online retailers in the presence of product value uncertainty and consumer anticipated regret. Journal of Industrial Engineering and Engineering Management. 2022; 36(05): 181-195.
23. Zhou JH, Li QQ, Xu XL. Pricing Strategy of Fashion Products with Strategic Consumer Regret. Chinese Journal of Management Science. 2022;30(11):117-126.
24. Shi BL, Xu Q, Sun ZM. Pre-sale and Return Strategy of Retailers Considering Consumer Regret Behavior. Operations Research and Management Science. 2022; 31(10): 40-46.
25. Wang XH, Zeng C. A model of advance selling with consumer heterogeneity and limited capacity. Journal of Economics.2016;117(2):137-165.
26. Xu J, Duan Y. Pricing, ordering, and quick response for online sellers in the presence of consumer disappointment aversion. Transportation Research Part E: Logistics and Transportation Review. 2020;137: 101925.

***Q3:*** *Finally, I'm not sure the reason but the title shown in your submission is in Chinese. You may check with the Journal's staff to make sure it's acceptable.*

**Answer3:** Thank you for your suggestions, we agree and have updated the content. We will correct the title when we submit this revision.
